# Level of anxiety versus self-care in the preoperative and postoperative periods of total laryngectomy patients**^1^**

**DOI:** 10.1590/1518-8345.0743.2707

**Published:** 2016-06-14

**Authors:** Clara Inés Flórez Almonacid, Alfredo Jurado Ramos, María-Aurora Rodríguez-Borrego

**Affiliations:** 2 PhD, Associate Professor, Departamento de Enfermería, Universidad de Córdoba, Córdoba, Andalucía, Spain.; 3 PhD, Full Professor, Departamento de Medicina, Universidad de Córdoba, Córdoba, Andalucía, Spain.; 4 PhD, Full Professor, Departamento de Enfermería, Universidad de Córdoba, Córdoba, Andalucía, Spain.

**Keywords:** Neoplasms, Laryngeal Mucosa, Laryngectomy, Anxiety, Adjustment Disorders, Activities of Daily Living

## Abstract

**Objective:**

estimate the prevalence of anxiety in laryngectomy patients in the pre and postoperative periods and its relation with the self-care level.

**Method:**

observational research of 40 patients with stage IV laryngeal cancer. Three observations took place: in the preoperative phase, at seven and at 14 days after the surgery; between June 2010 and December 2012. Two self-care levels were defined: self-sufficient and needing help for activities of daily living and treatment-related activities. To assess the anxiety levels, Zigmond's hospital anxiety scale (1983) was used.

**Results:**

in the preoperative and postoperative phases, the patients presented high levels of anxiety. Concerning self-care, on average, self-sufficient patients presented lower levels of anxiety than patients who needed help to accomplish activities of daily living and activities deriving from the surgery, without significant differences.

**Conclusion:**

anxiety is present at all times in laryngectomy patients and the reduction of the self-care deficit seems to decrease it, without putting a permanent end to it.

## Introduction

Laryngeal cancer is emotionally traumatic, due to the change in the body image and functional deterioration resulting from the cancer and its surgical treatment; considering that the patients fear a bad prognosis, the pain, the loss of dignity, physical disfigurement and worsening of communication; the loss of this function can provoke feelings of vulnerability, sadness and fear^1^. 

Anxiety, in turn, is an emotional response or response pattern that comprises cognitive aspects: unpleasant, tension and apprehension; physiological aspects characterized by a high degree of activation of the autonomous nervous system (ANS) and motor aspects, which tend to imply maladjusted and hardly adaptive behaviors^2^. The North American Nursing Diagnosis Association (NANDA) established a diagnostic label called Anxiety, defined as: "A vague, uneasy feeling of discomfort or dread accompanied by an autonomic response, with the source often nonspecific or unknown to the individual; a feeling of apprehension caused by anticipation of danger. It is an altering signal that warns of impending danger and enables the individual to take measures to deal with threat"^3^. 

Psychological morbidity is frequently underdiagnosed. While most patients are capable of facing their anguish and adapting to changes, some experience more anguish, anxiety and depression^1^. The prevalence of anxiety in head and neck cancer patients greatly varies; several authors have estimated that the prevalence of anxiety ranges between 5% and 87%^4^ ^-^ ^6^. 

Despite considerable advances in the surgical techniques designed to minimize the dysfunctional image and changed imaged in the postoperative phase, the results are traumatic for the patient^7^ ^-^ ^8^. 

 Some authors^1^ ^,^ ^9^ have demonstrated that the recovery after a head and neck cancer surgery can include long-term physical, emotional, social and behavioral debilitating sequelae, and that the anxiety can continue increasing linearly after the end of the treatment. 

Another aspect that stands out in total laryngectomy patients is that specific care is required, deriving from the surgery, which the patient needs to learn with a view to daily self-care. Laryngectomized patients experience different modifications, causing self-care needs and a self-care deficit, which should be solved or compensated to prevent complications, live with their disease and develop appropriately, incorporating self-care into their daily life as a fundamental tool to contribute to the maintenance of their health and improvement of their quality of life^10^ ^-^ ^11^. 

The analysis of the information described reveals a lack of research on the psychological consequences and self-care of laryngeal cancer patients during the preoperative and immediate postoperative phases. Although different studies have examined the psychosocial aspects of cancer, the death threat, body image problems, fears of treatment (surgery, radiation and chemotherapy), the family's reaction to the potential image change, social and professional problems, and psychological reactions like the laryngeal cancer patients' anxiety and depression during the chemotherapy/radiotherapy or during the rehabilitation, research in the immediate periods before and after the surgical treatment is scarce. Therefore, this study was undertaken in response to the question about whether patients with higher anxiety levels have a greater self-care deficit; and aiming to estimate the prevalence of preoperative and immediate postoperative anxiety in laryngectomized patients and its relation with the self-care level.

The reference framework in the care for these patients was: Orem's general theory, which consists of the Self-Care Theory (SCT), Self-Care Deficit Theory (SCDT) and Nursing System Theory (NST)^12^ ^-^ ^13^. 

The development of Orem's Self-Care Theory is ordered and systematic, offering a global assessment of the patient. In addition, it can be used in any cancer stage and defines the activity range of the nurse, the patient and the caregivers, with a view to satisfying the patient's Self-Care Demands^12^.

Self-Care (SC) is the set of actions a person performs to control internal or external factors, which can compromise that person's life and further development. Orem departed from the premise that all individuals are capable of satisfying their self-care needs^12^. The Self-Care Deficit (SCD) explains the relation between the person's abilities and power to achieve the objectives of SC. The self-care deficit does not refer to a specific limitation, but establishes the relation between what the individual is capable of and his/her needs. When the SCD is established, professional nursing interventions are made for some time to compensate for it; Orem calls this Therapeutic Self-Care Demand (TSCD)^12^ ^-^ ^13^. 

When a self-care deficit exists, the nurse is able to compensate for it through the Nursing System (NS), which can be: wholly compensatory, partly compensatory and supportive-educative^12^. 

## Method

Observational study of patients medically and surgically judged with total laryngectomy, with a final diagnosis of squamous cell epidermoid carcinoma (stage T4), during their hospital stay at the Clinical Otorhinolaryngology Service of a tertiary hospital in Southern Spain, between June 2010 and December 2012. The sample size was calculated to estimate a proportion in finite populations (based on the number of total laryngectomy patients in 2008-2009 hospitalized at the research hospital, corresponding to an average 45 persons). With a prevalence of 3.4% of laryngeal cancer in Spain, a 2% precision level and a 95% confidence level, the sample size was 40 cases. Sampling happened consecutively to the patient's internment for intervention at the research hospital.

The research variables were: preoperative anxiety [t_0_], at seven [t_1_] and 14[t_2_] days after the surgery, demographic data [gender, age, hospital stay], smoking habit [smoker, former smoker (having quit smoking one year before the start of the study) and non-smoker], alcohol consumption [drinker, former drinker (having quit drinking one year before the start of the study) and non-drinker], self-care level [self-sufficient, needs help with care activities: self-care deficit (yes-no) and support networks [yes-no].

To measure the anxiety, Zigmond's Hospital Anxiety and Depression Scale - HADS^14^ was used, developed to detect anxiety (anxiety subscale HADS-A) and depressive disorders at non-psychiatric hospital services, avoiding overlapping with symptoms caused by physical illness. This self-reported scale consists of 14 items, seven of which measure anxiety. The response format is a four-point Likert scale. One of the advantages is its short response time. In the scoring of the anxiety subscale, scores between 0 and 7 points are not considered as cases, scores between 8 and 10 as doubtful cases and between 11 and 21 as cases. The anxiety subscale (HADS-A) contains seven items, scored between 0 (never, normal) and 3 (continuous, very intense), considering scores of 11 or higher as "defined cases". Nevertheless, various studies use different cut-off points, referring that a lower cut-off point provides excellent properties to detect psychological problems^15^. In this study, it was considered that, at cut-off point superior to 8, the patients suffered from anxiety^15^. The anxiety subscale (HADS-A) was validated in the Spanish population, with a sensitivity ratio of 78% and a specificity ratio of 74%^16^. 

To value the support networks, the following were considered: whether the patient had a person for everything needed, occasionally, only for concrete things or no social networks.

For the self-care measure, Orem's theory was used. Two self-care levels were defined: self-sufficient and need help to accomplish activities (self-care deficits), operationally defined as the performance of activities of daily living (ADLs), assessed using Barthel's index^17^, a questionnaire that measures the patient's ability for independent self-care, with a high reliability coefficient (Cronbachs alpha 0.86-0.92). Its replicability is excellent, with weighted kappa correlation coefficients of 0.98 intra-observers and over 0.88 inter-observers, and international consistency (Cronbach's alpha 0.86-0.92)^18^. The Barthel index assesses ten activities of daily living: feeding, bathing, dressing, grooming, toileting, bowel control, bladder control, chair transfer, ambulation and chair climbing. The total score ranges between 0 and 100. Dependence is mild when the score ranges between 91 and 99, moderate between 61 and 90, severe between 21 and 60 and total for scores inferior to 20^17^. In this study, the patients were classified according to the score, as follows: self-sufficient if Barthel index 90-100; need help if Barthel index 21-90 and dependence if Barthel index inferior to 21. 

The self-care activities specifically related to the surgical procedure were grouped as independent or in need of help to perform them; the activities were assessed using the nursing outcomes classification, based on the outcome: acceptance of health condition, and the indicator: performs care deriving from the surgery: mobilization of tracheobronchial secretions (effective cough), stoma care (cure, change of tracheotomy cannula), sleep in half-seated position, communicate in writing and nasogastric tube feeding. Patients were classified as independent and in need of help to perform the activities^19^. 

On the day the patient was hospitalized for the surgical intervention, defined in the study as the pre-surgery (t_0_), the patient was interviewed to provide a detailed explanation on the purpose of the study and to request informed consent in writing; if the patient agreed, sociodemographic data, affiliation, disease and toxic antecedents were collected. The patient, in turn, completed the self-reported anxiety scale (HADS-A) and the self-reported self-care level for activities of daily living and surgery-related activities.

At seven (t_1_) and 14(t_2_) days post-surgery, the patient was again interviewed to revise the clinical history, assess the self-care level (self-sufficiency or help with activities of daily living and surgery related activities), the presence of family networks was reassessed and the patient completed the self-reporting. 

The research protocol received approval from the research ethics committee of the hospital center. The study was undertaken in accordance with the requirements formulated in the Helsinki Declaration (Seoul revision, October 2008) and Spanish law regarding the treatment, information and transfer of personal data of all participants, in compliance with the determinations in Organic Law 15/1999, dated December 13^th^, on the Protection of Personal Data and RD 1720/2007. 

In the statistical analysis, the patient's characteristics are described through frequencies and percentages for qualitative variables, or averages, standard deviations (SD), minima and maxima for quantitative variables. 

For the variables anxiety and self-care level, repeatedly measured in the same patients (preoperative, at seven and 14 days postoperative), the description and statistical analysis techniques used were techniques for repeated data. The quantitative variables are described as differences of means among the different times in the study (among t_o_-t_1_-t_2_), SD for the differences and 95% confidence intervals. The qualitative variables are described using frequencies and the change in the status of the category among the distinct study times. The bivariate relations between the qualitative variables of interest and the anxiety and self-care level variables were studied using contingency tables at each clinical time, as well as the chi-squared test or Fisher's exact test (in case the percentage of crosses with expected frequency inferior to five was higher than 20%). In addition, to study the variation or relation of the variables between the pre-surgery time and seven and 14 days post-surgery, the Generalized Estimation Equation (GEE) model was used. 

## Results

Among the 40 total laryngectomy patients studied, 38 (95%) were men. The mean age of all patients was 61.73 (SD 11.08) years, with a maximum age of 82 and a minimum age of 42 years; in men, the mean age was 61.8 years (SD 11.2) and, in women, the mean age was 59.0 (SD 8.48). The mean length of the hospital stay was 18.5 (SD 4.8) days, with a minimum age of 14 and a maximum age of 38; 100% of the patients indicated they had been informed about the surgery and its consequences.

In Table 1, the socioeconomic and clinical characteristics and antecedents of the patients in the preoperative period are displayed.

**Table 1:** Sociodemographic description and antecedents in the preoperative period of laryngectomy (n=40). Cordoba, CAA, Spain, 2014

Variables	Category	N	%
Gender	Man	38.0	95
	Woman	2.0	5
Education	Primary	28.0	70
	Secondary	7.0	17.5
	College	1.0	2.5
	Others	4.0	10
Marital Status	With partner*	29.0	72.5
	Separated	8.0	20
	Single	3	7.5
Professional Situation	Works	17	42.5
	No job	1	2.5
	Invalidity	3	7.5
	Retired	19	47.5
Anxiety	No	12	30.0
	Yes	28	70.0
Tobacco Consumption	Non-smoker	1	2.5
	Smoker	26	65
	Former smoker	13	32.5
Alcohol consumption	Drinker	25	62.6
	Non-drinker	10	25
	Former drinker	5	12.5
Support network	Yes	38	95
	No	2	5
Self-care level	Self-sufficient	34	85
	Help ADL**^+^**	6	15

*With partner (married, consensual union). ^+^ADL (Help for activities of daily living)

 Initially, the patients presented a mean score of 9.325 (SD 3.392) on the subscale of hospital anxiety (HADS-A), with a minimum of 4 and a maximum of 18. The patients entered the hospital feeling anxious and the anxiety level increased at seven days post-surgery, dropping almost to the baseline levels at 14 days post-surgery (Table 2).

**Table 2:** Frequency and Percentage of patients with anxiety (n=40). Córdoba, CAA, Spain, 2014

	Anxiety	P
Preoperative (t**_0_** )	28(70%)	
Postoperative 7 days (t**_1_** )	39 (97.5%)	p=0.001
Postoperative 14 days (t**_2_** )	29(72.5%)	p=0.001

On average, anxiety increased 2.175 points between the preoperative period and at seven days after the surgery. Between days seven and 14, anxiety dropped by an average 1.925. Overall, in the interval [t_0_, t_2_], anxiety increased by 0.25. Anxiety levels were higher between the preoperative phase and seven days post-surgery than between the seventh and 14^th^ day post-surgery (Table 3).

**Table 3:** Difference in anxiety between preoperative phase and at seven and 14 days after laryngectomy surgery (n=40). Cordoba, CAA, Spain, 2014

	Mean	SD	Confidence interval 95%	
			Inferior	superior
Differences between t**_0_** and t**_1_** (t**_0_** - t**_1_** )	-2.17	2.65	-3.02	-1.32
Differences between t**_1_** and t**_2_** (t**_1_** -t**_2_** )	1.92	2.16	1.23	2.61
Differences between t**_0_** and t**_2_** (t**_0_** -t**_2_** )	-0.25	3.44	-1.35	1.35

Anxiety statistical significance between (t_0_- t_1_) p=0.001 and between (t_1_ -t_2_) p-=0.002

The variable anxiety was not statistically related with gender, age, professional situation, tobacco consumption, alcohol, functional level and presence of family network. Concerning the self-care level, when they were hospitalized, 85% of the patients were self-sufficient for activities of daily living. Seven days after the surgery, however, 97.5% needed help with meals, mobilization of secretions, care for the wound and laryngectomy cannula, communication, mobilization and activity. At 14 days post-surgery, 70% still needed help to change the cannula (Table 4).

**Table 4:** Frequency and percentage of patients' functional level in the pre- and postoperative period of laryngectomy. (n=40), Cordoba, CAA. Spain 2014

	Preoperative	Postoperative			P
		7 Days	14 Days	
Self-sufficient	34(85%)	1 (2.5%)	12(30%)	<0.0001*
Needs help	6(15%)	(97.5%)	8(70%)	<0.0001**^†^**

Statistical significance between [t_0_-t_1_]

* p <0.0001 between [t_1_-t_2_]

† p<0.0001

**Figure 1:**
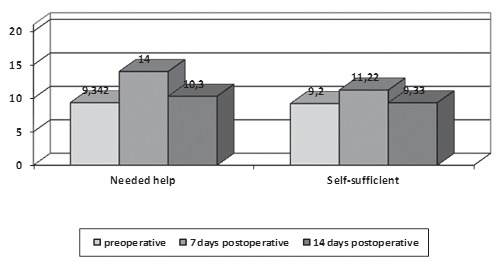
Self-care and anxiety in preoperative and postoperative periods of laryngectomy (n=40). Cordoba, CAA, Spain, 2014

In the preoperative phase, 71.4% (n=25) of the patients were self-sufficient for activities of daily living (ADL) and presented anxiety. At seven days post-surgery, 97.2 % (n=35) needed help for self-care such as hygiene, mobilization of tracheobronchial secretions (airway aspiration), change of tracheotomy cannula, written communication and nasogastric tube feeding and still presented anxiety; similarly, at 14 days post-surgery, 68.9% (n=21) needed help with tracheotomy care and still experienced anxiety, although less than after seven days. On average, self-sufficient patients presented lower anxiety levels than patients who needed help to accomplish activities of daily living and surgery-related activities, without significant differences (Figure 1).

## Discussion

These study results should be taken with caution, as the sample size limits the weight of the findings; on the other hand, the scarce research on anxiety levels in the immediate postoperative phase of total laryngectomy support their publication.

Some studies^7^ ^-^ ^9^ have demonstrated that the prevalence of anxiety in cancer patients in general is high. Nevertheless, there are great prevalence differences, with figures ranging from 5% to 87%; this range can be due to methodological differences, such as the use of different tools, different cut-off points, cancer staging, different types of cancer diagnoses or the phase in which the assessment took place. Although apparently contradictory, some studies, including meta-analyses, have demonstrated that, in comparison with a general population, the prevalence of anxiety in cancer patients is lower^5^ ^-^ ^6^.

This study has evidenced that, in the preoperative period, patients' anxiety level is high and neither drops nor rises distinctly in the postoperative period. The high prevalence of preoperative anxiety found was similar to recent studies^8^ ^,^ ^20^. Nevertheless, in other studies, the prevalence is low, without surpassing 50%^21^ ^-^ ^22^. 

Concerning the presence of postoperative anxiety, there is a lack of studies, generally involving radiotherapy patients; in most studies, fixed-term measures were used that do not consider the variation of time and its effects. 

In this study, the increased anxiety was not statistically related with any of the variables studied. Nevertheless, it is clinically relevant that patients who presented preoperative anxiety, probably conditioned by the diagnosis, displayed even higher anxiety levels at seven days postoperative, with a reduction in self-care activities; at 14 days postoperative, on the other hand, anxiety levels had dropped and self-care activities had increased. This seems to be in accordance with authors^10^ ^-^ ^11^ who indicate that anxiety decreased in surgical patients with head and neck cancer at a specific point in time (fifth day postoperative). The increase in personal care, then, precedes the drop in anxiety levels. Self-care and anxiety have been significantly correlated on the fourth and fifth days after the surgery, with a relation between anxiety and self-care; a higher level of self-care corresponds to a lower level of anxiety. 

During the first postoperative week, most patients need help for ADLs, have no voice, experience communication problems, with the presence of the laryngectomy cannula and abundant secretions, neck edema, drainage, parenteral fluids and appearance of mucus plugs that obstruct the cannula, causing a feeling of lack of air. At 14 days, however, the levels of anxiety and help for ADLs dropped, the patients had returned to their daily self-care and had gained abilities for most surgery-related self-care, except care for the laryngectomy cannula, for which six out of ten patients needed help. 

This study reveals that the laryngeal cancer diagnosis causes anxiety. Nevertheless, it seems that self-care contributes to reduce anxiety levels. The high prevalence of anxiety found in the preoperative and postoperative phases in laryngectomized patients suggests that, in care for this kind of patients, a multiprofessional approach is needed to appropriately attend to the complex psychological needs of these patients and their families.

## Conclusion

These study results on the prevalence of anxiety in the preoperative and immediate postoperative periods in laryngectomized patients and its relation with self-care levels indicate that anxiety is present at all times in total laryngectomy patients.
